# Successful Substrate-Guided Ablation of Ventricular Tachycardia Storm in Acute Heart Failure Without Mechanical Circulatory Support

**DOI:** 10.1016/j.jaccas.2025.106214

**Published:** 2025-12-03

**Authors:** Le Uyen Phuong Tran, Quoc Hoang Nguyen, Cao Dat Tran, Ngoc Dung Kieu, Chau N. Vo

**Affiliations:** a Section of Cardiac Electrophysiology, Cho Ray Hospital, Ho Chi Minh City, Vietnam; b Section of Cardiac Electrophysiology, Medical University of South Carolina, Charleston, South Carolina, USA

**Keywords:** acute heart failure, systolic heart failure, ventricular tachycardia

## Abstract

**Background:**

Ventricular tachycardia (VT) storm in advanced systolic heart failure carries high morbidity and mortality. Ablation is often performed with advanced imaging and mechanical circulatory support, which may not be available in resource-limited settings.

**Case Summary:**

A 62-year-old man with ischemic cardiomyopathy (left ventricular ejection fraction: 25% to 30%) presented with recurrent monomorphic VT refractory to antiarrhythmic therapy, complicated by hemodynamic instability and multiorgan dysfunction. Mechanical circulatory support was not feasible. Substrate-guided mapping using voltage, late potentials, local abnormal ventricular activity, isochronal late activation mapping, and decrement-evoked potential mapping localized a critical isthmus in the basal posterior left ventricle. Ablation terminated VT and rendered it noninducible under monitored anesthesia care with norepinephrine and low-dose dobutamine.

**Conclusion:**

Functional substrate mapping with limited intra-VT mapping and tailored anesthesia enabled procedural stability and successful VT ablation.

**Take-Home Message:**

Substrate-guided mapping with individualized hemodynamic support can achieve safe, effective VT ablation in resource-limited settings.

## Visual Summary

**Figure:**
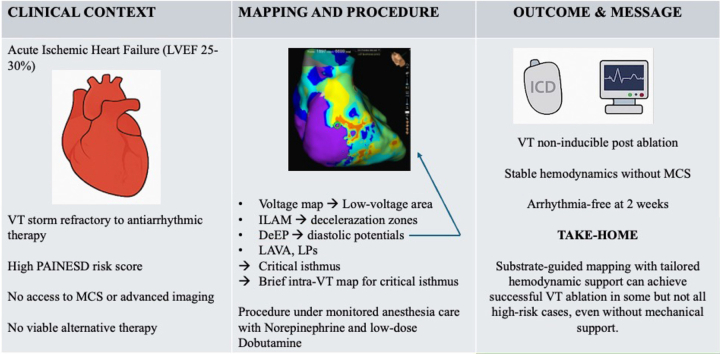

Ventricular tachycardia (VT) storm in patients with structural heart disease is a life-threatening condition associated with high morbidity and mortality. Although antiarrhythmic drugs and implantable cardioverter-defibrillator therapies are first-line treatments, catheter ablation has emerged as an effective strategy for refractory cases.^1^^,^^2^ Optimal ablation requires integration of advanced imaging, meticulous substrate characterization, and, in high-risk patients, mechanical circulatory support (MCS).^3^^,^^4^ However, in many developing countries, these resources are not always available, creating significant challenges in patient management.

**VISUAL SUMMARY:**
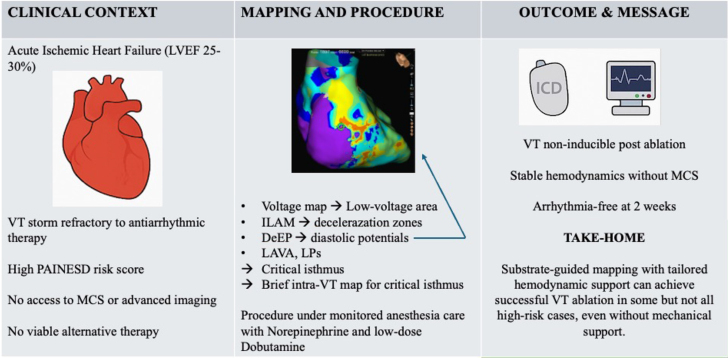
Successful Substrate-Guided VT Ablation Without Mechanical Support in Acute Heart Failure

We present a case of successful substrate-guided ablation of VT storm in advanced ischemic cardiomyopathy without access to MCS or advanced imaging.

**Take-Home Message**

• Substrate-guided functional mapping strategies, including voltage, ILAM, DeEP, LAVA, and late potentials analysis, can enable safe and effective VT ablation, in some cases even without advanced imaging or mechanical circulatory support. Individualized anesthetic and hemodynamic management are essential to procedural stability in high-risk patients, offering a practical framework for centers operating in resource-limited environments.

## History of Presentation and Past Medical History

**Figure 1:**
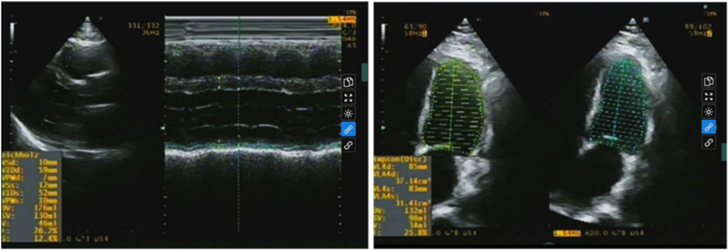
Transthoracic Echocardiography Apical 4-chamber view showing dilated left ventricle with global hypokinesis and reduced left ventricular ejection fraction (25% by Simpson biplane method).

A 62-year-old man with hypertension and type 2 diabetes mellitus was referred with recurrent monomorphic VT storm refractory to amiodarone and lidocaine. He had suffered an acute myocardial infarction 2 months prior, with failed revascularization of the proximal dominant right coronary artery, leading to ischemic cardiomyopathy (left ventricular ejection fraction: 25% to 30%, NYHA functional class III) (Figure 1).

**Figure 2:**
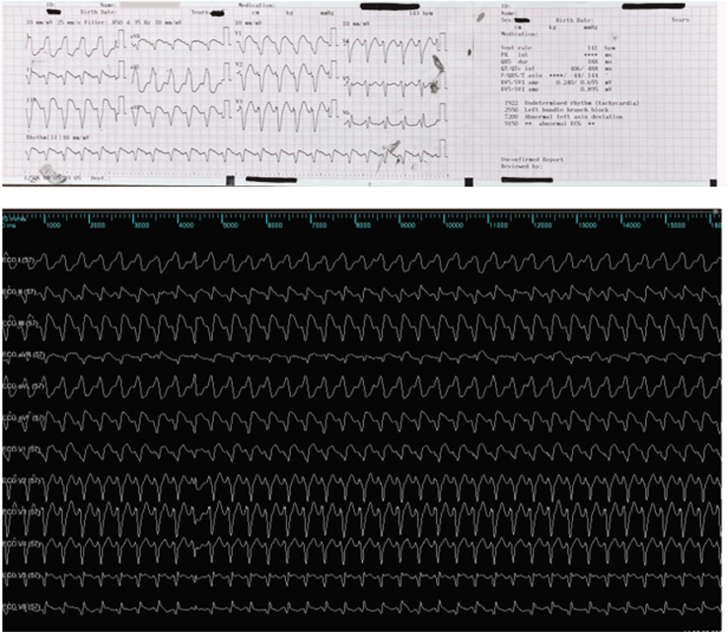
ECG Demonstrating Ventricular Tachycardia 12-lead ECG demonstrating monomorphic ventricular tachycardia at 141 beats/min with left bundle branch block–like morphology, left axis deviation, and QRS width 188 ms with intermittent capture beat. ECG = electrocardiogram.

At presentation, he was in sustained monomorphic VT requiring multiple cardioversions (Figure 2). His blood pressure was 100 to 110/50 to 60 mm Hg.

## Investigations

**Figure 3:**
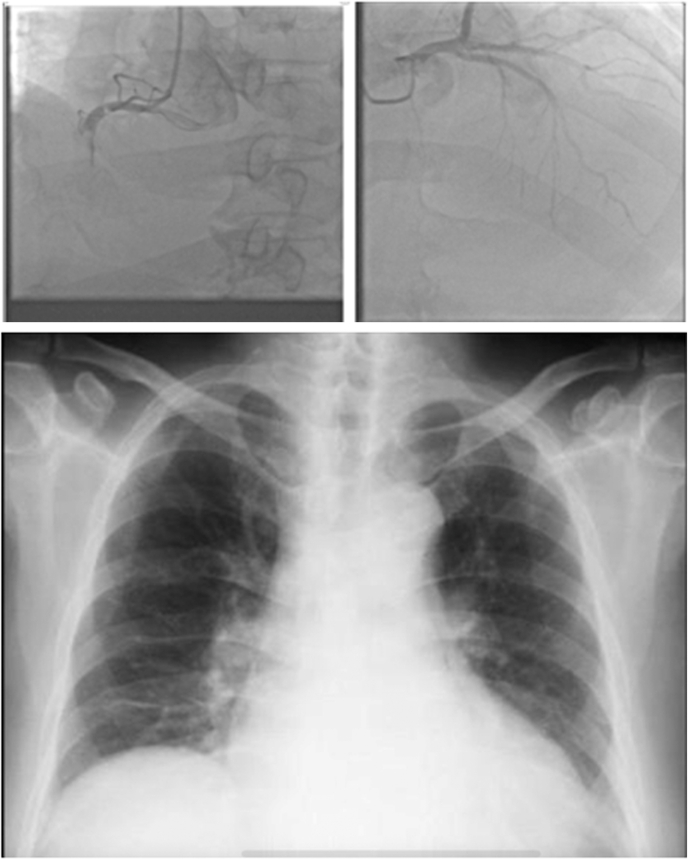
Preprocedural Coronary Angiography and Chest X-Ray

Laboratory tests revealed acute kidney injury (creatinine: 1.5 mg/dL), mild transaminitis, elevated N-terminal pro–B-type natriuretic peptide (27,199 pg/mL), lactate 1.8 mmol/L, and stable troponin elevation (279 ng/L). Chest x-ray showed mild pulmonary congestion (Figure 3). His PAINESD score was 23, indicating high risk of intraoperative hemodynamic decompensation.

## Management (Electrophysiology Study and Ablation)

Urgent ablation was undertaken for ongoing VT storm and clinical deterioration, with no viable alternative therapy. Given the high-risk PAINESD score and unavailability of MCS, the procedure was conducted under monitored anesthesia care with propofol, midazolam, norepinephrine, and low-dose dobutamine (5 μg/min). Intraoperative VT was temporized by additional lidocaine bolus at the beginning of the procedure, ventricular programmed override pacing, and external cardioversions. Intraoperative arterial blood gas and lactate levels were closely monitored and remained within normal limit range.

**Figure 4:**
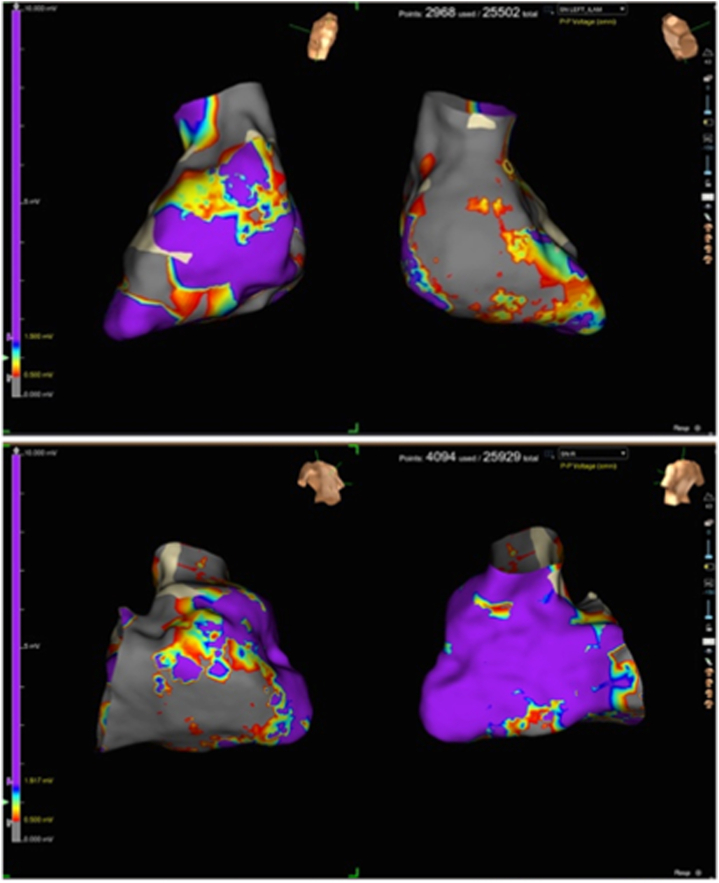
Right and Left Ventricular Bipolar Voltage Map Proximal dominant RCA occlusion on coronary angiography correlated with a dense scar area (red) at the basal posterior septum on the endocardial voltage map of the left ventricle during sinus rhythm. RCA = right coronary artery.

**Figure 5:**
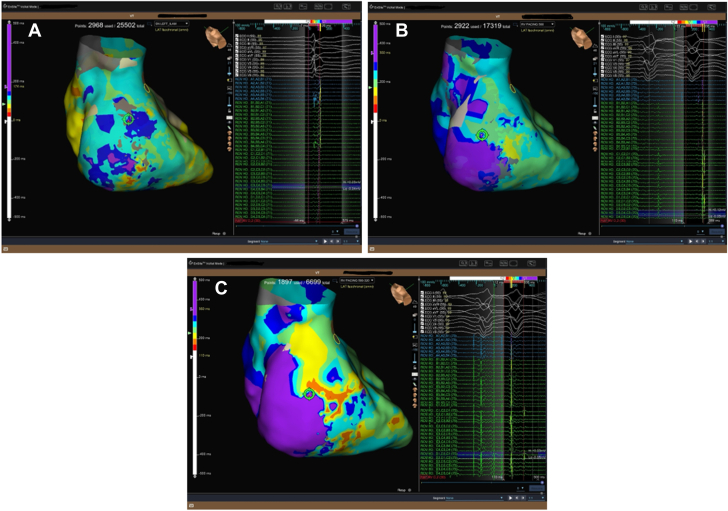

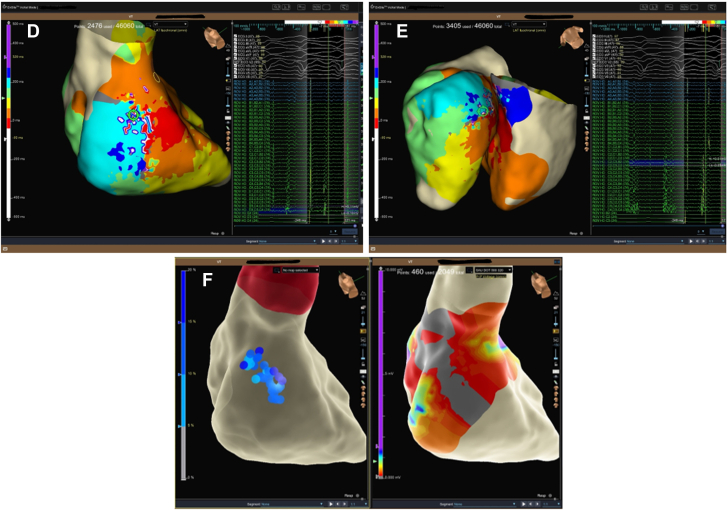
Isochronal Late Activation Mapping (A) ILAM in sinus rhythm detected deceleration zones (DZs) at the posterobasal wall of the left ventricle. (B) ILAM with RV pacing at cycle length 500 ms detected diastolic potentials corresponding to the entrance of the VT isthmus. (C) ILAM with S2 RV pacing at cycle length 500 to 320 ms (VERP + 20 ms) detected diastolic potentials corresponding to the entrance of the VT isthmus. (D) Local activation map during VT detected a mid-diastolic signal in the same area as with ILAM with S1 and S2 RV pacing. (E) Biventricular activation mapping during VT showed the RV was only passively activated via breakout into the RV posterior basal septum. (F) Ablations terminated the VT, and the postablation map showed a consistent block over that line. ILAM = isochronal late activation mapping; RV = right ventricular; VERP = ventricular effective refractory period; VT = ventricular tachycardia.

Electroanatomic mapping (EnSite X, Abbott) revealed a large low-voltage area in the basal posterior septal left ventricular wall (Figure 4). Isochronal late activation mapping (ILAM) identified deceleration zones, and decrement-evoked potential (DeEP) mapping unmasked late diastolic potentials with unidirectional block in the same area (Figures 5A to 5F). Clinical VT occurred spontaneously (left bundle branch block morphology, superior axis, cycle length of 410 ms) (Figure 1). Local activation mapping confirmed the critical isthmus at the basal posteroseptal left ventricle, with passive exiting to the basal right ventricular septum (Figure 5F, Video 1). Limited entrainment attempt was performed from the mid-diastolic potential signals but could not capture the myocardium. To prevent hemodynamic decompensation, no further entrainment was attempted.

Radiofrequency ablation at the mid-diastolic signals slowed and terminated VT within 30 seconds (Video 2). Additional substrate modification was performed, targeting the deceleration zones, late potentials, and local abnormal ventricular activity (LAVA). Postablation programmed ventricular stimulation on 600 ms and 400 ms drivetrains with triple extra stimuli could not reinduce VT.

The patient tolerated the 3-hour procedure without escalation of hemodynamic support. An implantable cardioverter-defibrillator was placed 2 days later. He was discharged without antiarrhythmic therapy.

## Follow-Up

At the 2-week follow-up, the patient remained hemodynamically stable and free of arrhythmia recurrence.

## Discussion

Catheter ablation for VT storm is challenging in patients with advanced systolic heart failure. Optimal preparation includes advanced imaging and readiness for MCS, but these may be unavailable in resource-limited settings.^5^^,^^6^

In the current case, functional substrate mapping strategies, including voltage, ILAM, DeEP, late potentials, and LAVA, allowed precise identification of the arrhythmogenic substrate despite limited mapping in VT. These methods have been shown to improve ablation outcomes beyond conventional static scar mapping.^7^, ^8^, ^9^, ^10^, ^11^

Anesthetic management was tailored to avoid hemodynamic collapse. General anesthesia may worsen instability, whereas moderate to deep sedation with vasopressors is supported in selected cases.^3^ Low-dose dobutamine, although rarely reported, provided additional inotropic support without precipitating arrhythmias.

This case illustrates that systematic substrate-guided ablation with careful anesthetic planning can be successfully applied even when advanced imaging and mechanical support are unavailable. However, this substrate-guided approach may not be feasible or safe in patients with incessant VT or severe cardiogenic shock, in whom MCS is essential for procedure stability and success.

## Conclusions

In patients with VT storm and acute systolic heart failure, successful ablation can be achieved in resource-constrained settings through functional substrate-guided mapping and tailored anesthetic management. This approach may serve as a practical strategy when advanced imaging and mechanical circulatory devices are not accessible.

**Equipment List:** 

• Electroanatomic mapping system: EnSite X (Abbott)
• Ablation catheter: TactiCath contact force–sensing catheter (Abbott)
• Mapping catheter: Advisor HD grid mapping catheter (Abbott)
• Recording system: EnSite precision recording and navigation platform (Abbott)
• Ablation generator: Ampere RF generator (Abbott)
• Imaging: standard fluoroscopy (Siemens Artis Zee system)
• Intracardiac echocardiography: AcuNav (Siemens Healthineers)
• Anesthesia monitoring: IntelliVue MX750 patient monitor (Philips)
• Medications: propofol, midazolam, norepinephrine, dobutamine, lidocaine
• Vascular access: standard femoral venous and arterial sheaths (8 F-8 F)
• Defibrillation/external cardioversion: HeartStart MRx defibrillator (Philips)
• Implantable cardioverter-defibrillator: Gallant HF (Abbott)

### Learning Objectives

• To recognize that substrate-guided functional mapping strategies (ILAM, DeEP, late potentials, LAVA) can facilitate VT ablation with limited intra-VT mapping.
• To understand that norepinephrine with low-dose dobutamine under monitored anesthesia care may provide adequate hemodynamic support in select high-risk VT ablation cases when MSC is unavailable.
• To highlight strategies for tailoring VT ablation in resource-constrained environments.

## Funding Support and Author Disclosures

The authors have reported that they have no relationships relevant to the contents of this paper to disclose.

## Appendix

Video 1 Activation Map During VT Confirming Re-entry Mechanism Within the Basal Posterior Septum LV but Breakout Toward the RV Septum

Video 2 Ablation Site and Acute Success Radiofrequency ablation at the basal posterior LV septum successfully terminated the VT within 30 seconds. Postablation programmed stimulation failed to reinduce any VT.
